# Performance of the SARC-F (Strength, Assistance Walking, Rise From a Chair, Climb Stairs, and Falls) Questionnaire for Sarcopenia Screening in Patients With End-Stage Renal Disease on Hemodialysis

**DOI:** 10.7759/cureus.97705

**Published:** 2025-11-24

**Authors:** Edgar Dehesa-Lopez, Donají Ramírez-Sosa, Sarahy Sosa-Guerrero, Fatima Lopez-Moreno, Mónica Fernanda Curiel-González, Estefanía Araujo-Rocha

**Affiliations:** 1 Nephrology, Universidad Autónoma de Sinaloa, Culiacán, MEX; 2 Internal Medicine, Universidad Autónoma de Sinaloa, Culiacán, MEX

**Keywords:** chronic kidney disease (ckd), hd (hemodialysis), sarc-f questionnaire, screening, sarcopenia

## Abstract

Introduction: The systematic screening of sarcopenia among patients with end-stage renal disease (ESRD) on hemodialysis (HD) has emerged as a critical component of comprehensive patient assessment and management. The SARC-F (Strength, Assistance walking, Rise from a chair, Climb stairs, and Falls) is a simple and widely used tool for sarcopenia screening across multiple populations; however, its diagnostic performance in HD remains uncertain. We aimed to evaluate the diagnostic performance of the SARC-F questionnaire for sarcopenia screening in HD patients.

Methods: Cross-sectional diagnostic accuracy study in Mexican patients with ESRD on HD. Sarcopenia was defined according to European Working Group on Sarcopenia in Older People 2 (EWGSOP2) criteria: appendicular muscle mass, handgrip strength, and gait speed. The Spanish version of the SARC-F validated for Mexico was used; cut-off >4 points. Sensitivity, specificity, predictive values, receiver operating characteristic (ROC) curve, and area under the curve (AUC) were estimated. Results: Ninety-four patients were included (62.8% men; mean age 54±14 years). Sarcopenia prevalence was 8.5%. SARC-F >4 was observed in 17%. Diagnostic performance was poor: AUC 0.40 (95%CI 0.16-0.63; p=0.35), sensitivity 13% (95%CI, 2.2-47.1%), specificity 83% (95%CI, 73.2-89.1%), positive predictive value 6% (95%CI, 1.1-28.3%), and negative predictive value 91% (95%CI 82.6-95.6%).

Conclusions: SARC-F showed poor diagnostic performance in HD patients. Its high specificity and negative predictive value suggest utility to rule out sarcopenia; combining SARC-F with objective measures of muscle mass and performance is recommended for more accurate screening.

## Introduction

Sarcopenia is a progressive and generalized skeletal muscle disorder characterized by decreased muscle mass, strength, and function, which is associated with an increased risk of adverse outcomes such as falls, fractures, physical disability, and mortality [1]. Although initially considered a pathology exclusive to the geriatric population, it is now recognized as a clinical problem in chronically ill patients, including those with chronic kidney disease (CKD) [2,3].

Sarcopenia in patients undergoing dialysis has a complex and multifactorial etiology that involves both general and dialysis-specific factors. Key contributors include chronic inflammation, oxidative stress, metabolic acidosis, hormonal disturbances, and uremia-related protein-energy wasting, as well as the physical and psychological burden associated with dialysis therapy [3].

According to systematic reviews and meta-analyses, the pooled global prevalence of sarcopenia among dialysis patients ranges from 25.6% to 28.5% [3-6] and may vary between 10.9% and 29.7% depending on the diagnostic criteria applied and geographic regions [4]. Prevalence is higher in patients undergoing hemodialysis (HD) (26.8%) compared with those on peritoneal dialysis (17.5%) [6].

The clinical relevance of sarcopenia in patients undergoing HD stems from its high prevalence and its association with adverse outcomes, including impaired physical performance, frailty, increased rates of hospitalization, and mortality [3,5-7]. Individuals with sarcopenia exhibit a 1.9- to 6.9-fold higher risk of death compared with HD patients without sarcopenia, even after adjustment for clinical, biochemical, and comorbidity-related confounders [5,8,9]. Consequently, the early identification of sarcopenia is an essential component of the comprehensive evaluation and clinical management of this vulnerable population [3].

The SARC-F questionnaire is a simple and efficient screening tool that facilitates its integration into clinical practice [1,10,11]. This instrument assesses patient perception through five items covering Strength, Assistance in walking, Rising from a chair, Climbing stairs, and Falls [12]. Although SARC-F is the most widely used tool and is recommended by international societies for sarcopenia screening [1,10], evidence in patients on HD is limited, and it has shown low sensitivity and high specificity as a screening tool for sarcopenia in this population [13]. This highlights the need for additional clinical studies to evaluate its accuracy in this specific population.

The aim of our study was to evaluate the area under the curve, sensitivity, specificity, and positive and negative predictive values of the SARC-F questionnaire for the detection of sarcopenia in patients with ESRD on HD, using the European Working Group on Sarcopenia in Older People 2 (EWGSOP2) criteria as the reference standard, given that these are currently the most widely adopted criteria in clinical practice.

## Materials and methods

This was a cross-sectional diagnostic accuracy study conducted at Hospital Ángeles de Culiacán, Culiacán, Sinaloa, Mexico, in accordance with the ethical principles of the Declaration of Helsinki and the STARD (STAndards for Reporting Diagnostic accuracy studies) guideline for reporting transparency. The protocol was approved by the Ethics Committee, Centro de Investigación y Docencia en Ciencias de la Salud (CIDOCS), with registration number 180-2024. 

Study population

Patients included were over 18 years of age, of both sexes, diagnosed with ESRD on HD at the Hospital Ángeles de Culiacán, between July 1, 2024, and September 30, 2024, who accepted to participate and signed the informed consent. The sample size was one of convenience, including all eligible patients who consented to participate during the study period. Patients with active oncological disease, active infection, inability to walk, defined as the inability to perform the 4-meter walking test, and those with a contraindication for bioimpedance study were excluded. Patients for whom it was not possible to collect all variables were excluded.

**Figure 1:**
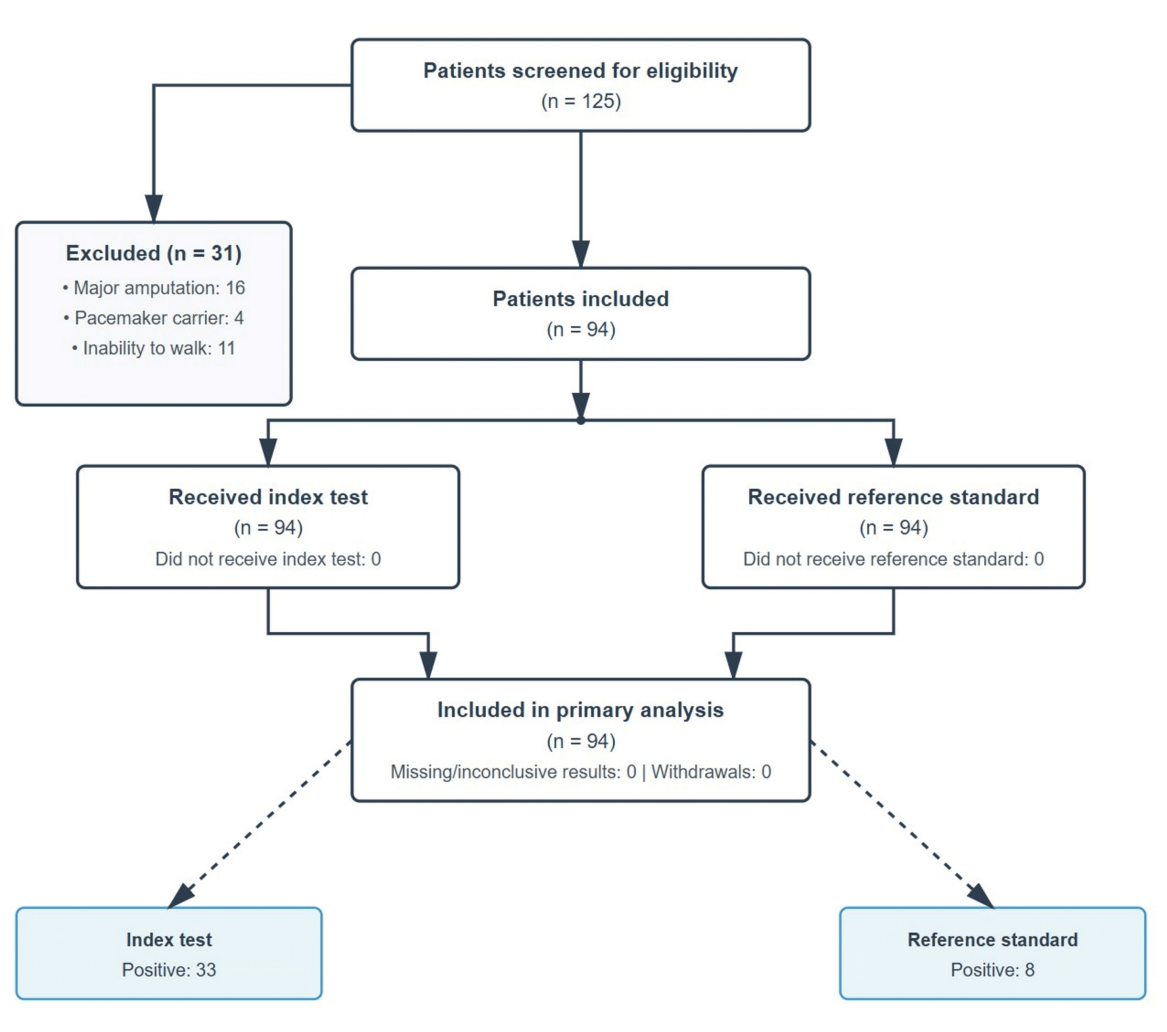
STARD flow diagram STARD: STAndards for Reporting Diagnostic accuracy studies

A total of 125 CKD patients on HD were evaluated, of which 31 were excluded (16 lower limb amputations, four pacemaker carriers, and 11 unable to complete the walk). A total of 94 patients were included in the final analysis (Figure 1). 

Definition of sarcopenia (reference standard) 

Sarcopenia was diagnosed according to the diagnostic approach and criteria of the EWGSOP2. Sarcopenia was considered present if the patient met the criterion for appendicular skeletal muscle mass index (ASMMi) and the criterion for muscle strength or gait speed.

The cut-off points used were handgrip strength (HGS) <27 kg in men and <16 kg in women; Appendicular Skeletal Muscle Mass Index (ASMMi) < 7 kg/m^2^ in men and <5.5 kg/m^2^ in women; and 4 m gait speed < 0.8 m/s [1].

HGS measurement was performed before the HD session. The Smedley III T19 analog manual dynamometer (Takei Kiki Kogyo Co., Ltd., Niigata, Japan) was applied to the dominant hand or the contralateral hand if the patient had an arteriovenous fistula. Whole-body resistance and reactance were measured after the HD session using the Quantum II Body Composition Analyzer (RJL Systems Inc., Michigan, United States) at 50 kHz. The obtained resistance and reactance parameters were entered into the BC4.0 software (Scooter Software, Inc., Madison, Wisconsin, United States) provided by the manufacturer for the calculation of each patient's body composition.

Appendicular muscle mass (AMM) estimation was obtained using the formula validated by Sergi et al. [14]: 

AMM (kg) = −3.964 + (0.227*talla2) + (0.095*peso) + (1.384*sex) + (0.064*Xc) 

ASMMi was calculated as AMM/height (Kg/m^2^). Physical performance was evaluated using the 4-meter walking test after the HD session. Four meters in length was delimited with a mark on the floor in an area of the HD clinic. Patients were instructed to walk at their usual pace, wearing their regular footwear, until crossing the designated line. Timing commenced upon a verbal start command. The use of assistive devices such as a cane or walker was permitted.

SARC-F questionnaire (index test) 

The SARC-F questionnaire [12] was administered by one of the investigators after completion of the HD session. The instrument consists of five items that assess strength, need for assistance in walking, difficulty rising from a chair, climbing stairs, and history of falls. For the first four items, perceived difficulty is scored as 0 (none), 1 (some), or 2 (a lot or unable). For the falls item, patients were asked: “How many times have you fallen in the past year?” with response options scored as 0 (none), 1 (1-3 falls), or 2 (>4 falls). The total SARC-F score was calculated as the sum of the five item scores. We used the Spanish version of SARC-F, adapted and validated for Mexico [15]. A total score >4 was considered indicative of sarcopenia risk and served as the diagnostic cutoff for evaluation [1].

Blinding

The application of the index test (SARC-F) was performed before the evaluation of the EWGSOP2 criteria, ensuring the SARC-F evaluator was blinded to the results of strength, mass, or gait speed. The assessment of muscle strength, gait speed, and the bioimpedance analysis was conducted by two different investigators who were blinded to the SARC-F questionnaire results.

Statistical analysis 

The Kolmogorov-Smirnov test was used to assess the normality of the data distribution. Descriptive statistics with means/standard deviations were used for continuous variables, and proportions were used for categorical variables. Comparisons between groups were performed with Student’s t-test for independent groups in the case of continuous variables and with the χ2 test for proportions. The area under the curve (AUC) for the SARC-F scores, defined according to the EWGSOP2 diagnostic criteria, was calculated, and receiver operating characteristic (ROC) curve analysis was conducted. The sensitivity, specificity, and positive and negative predictive values of SARC-F for the diagnosis of sarcopenia were calculated through contingency tables. Data analysis was performed with IBM SPSS Statistics for Windows, version 23 (Released 2015; IBM Corp., Armonk, New York, United States).

## Results

General characteristics of the study population 

A total of 94 patients were included in the study. The average age was 54 ± 14 years, with the male sex being the most frequent at 62.8% (n=59). The most frequent comorbidities were hypertension (81.9%, n = 77) and diabetes mellitus (52.1%, n=49). The main causes of CKD were diabetes mellitus (50%, n = 47) and arterial hypertension (30.9%, n = 29). Arteriovenous fistula was the most used vascular access (52.1%, n = 49), and the average time on HD was 4±3 years (Table 1).

**Table 1:** General characteristics of the study population. CKD: chronic kidney disease

Variables	Frequency	Percentage
Sex		
Female	35	37.2%
Male	59	62.8%
Comorbidities		
Diabetes Mellitus 2	49	52.1%
Hypertension	77	81.9%
Cause of CKD		
Diabetes Mellitus	47	50.0%
Hypertension	29	30.9%
Glomerulopathies	4	4.3%
Polycystic Kidney Disease	3	3.2%
Others	11	11.7%
Vascular Access		
Arteriovenous Fistula	49	52.1%
Catheter	45	47.9%
Years on hemodialysis	4	3

Frequency of sarcopenia in the study population 

According to the EWGSOP2 diagnostic criteria, sarcopenia was documented in 8.5% (n=8) of the patients. Of the sarcopenia cases, 12.5% (n=1) corresponded to a case of severe sarcopenia. No statistically significant differences were observed in the prevalence of sarcopenia between male and female sexes (8.5% vs 8.6%; p = 1.0). Regarding the individual criteria for sarcopenia, the gait speed criterion was more frequent in female patients (51.4% vs 20.3%; p = 0.003) (Table 2).

**Table 2:** Frequency of sarcopenia and EWGSOP2 individual criteria. Diagnostic criteria for sarcopenia according to the EWGSOP 2019 [1]; Spanish version of the SARC-F adapted and validated for Mexico [15] The comparison between groups was performed using the X^2^ test. EWGSOP2: European Working Group on Sarcopenia in Older People; SARC-F: Strength, Assistance walking, Rise from a chair, Climb stairs, and Falls

Variables	Total		Female		Male		p / X^2^ value
	Frequency	Percentage	Frequency	Percentage	Frequency	Percentage	
SARC-F Score							
SARC-F < 4	78	83.0%	26	74.3%	52	88.1%	0.09 (X^2^ = 2.98)
SARC-F > 4	16	17.0%	9	25.7%	7	11.9%	
Sarcopenia EWGSOP2 Criteria							
Without Sarcopenia	86	91.5%	32	91.4%	54	91.5%	1.0 (X^2^ =0.00)
With Sarcopenia	8	8.5%	3	8.6%	5	8.5%	
Handgrip Strength Criterion							
Normal handgrip strength	43	45.7%	17	48.6%	26	44.1%	0.83 (X^2^ = 0.18)
Reduced handgrip strength	51	54.3%	18	51.4%	33	55.9%	
Apendicular Muscle Mass Criterion							
Normal Appendicular Muscle Mass	82	87.2%	31	88.6%	51	86.4%	1.0 (X^2^ = 0.09)
Low Appendicular Muscle Mass	12	12.8%	4	11.4%	8	13.6%	
Gait Speed Criterion							
Speed > 0.8 m/s	64	68.1%	17	48.6%	47	79.7%	0.003 ( X^2^ = 9.77)
Speed < 0.8 m/s	30	31.9%	18	51.4%	12	20.3%	

Diagnostic performance of the SARC-F questionnaire in the study population 

**Figure 2:**
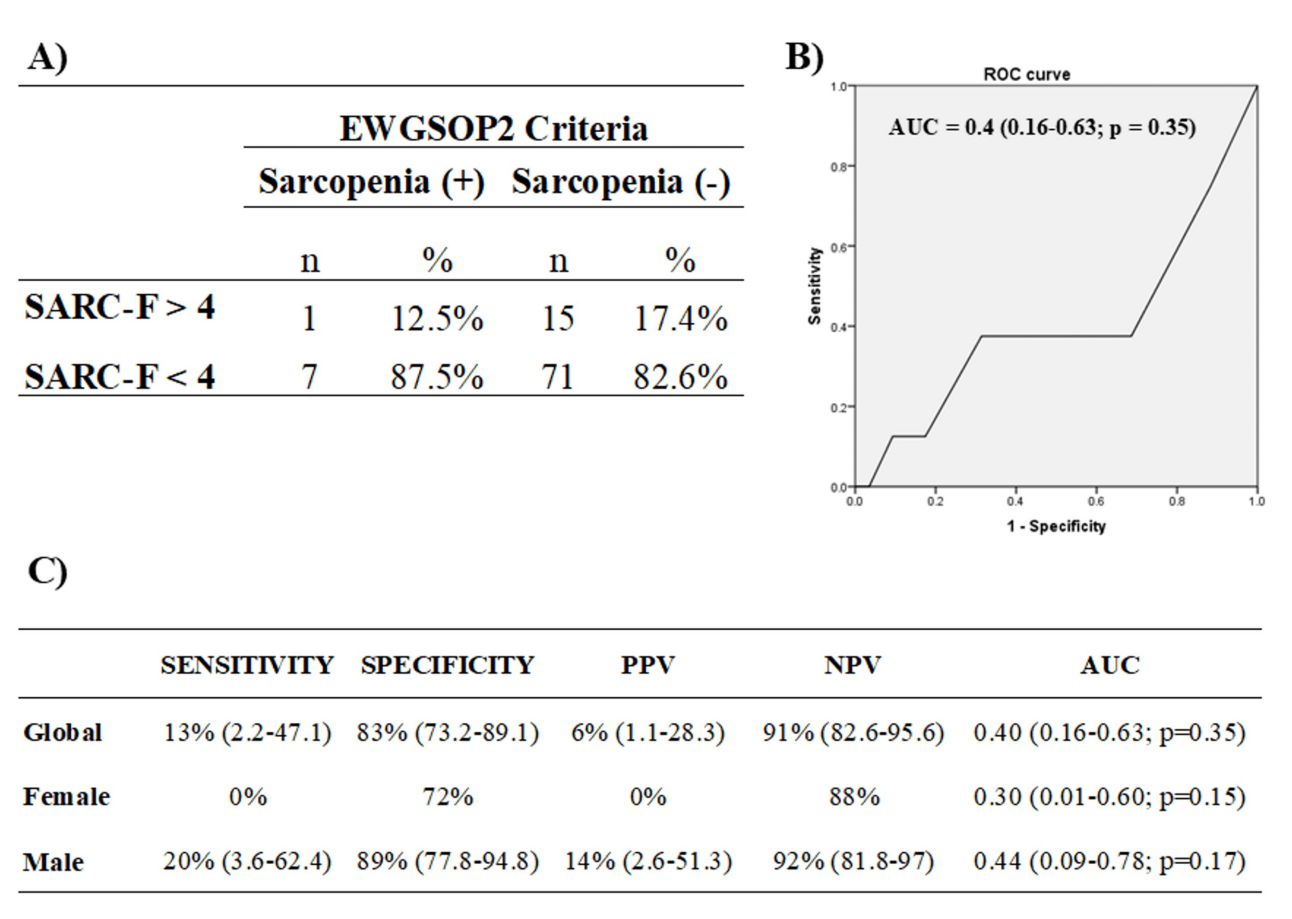
Diagnostic performance of the SARC-F questionnaire for sarcopenia screening. Diagnostic criteria for sarcopenia according to the EWGSOP2 [1]; Spanish version of the SARC-F adapted and validated for Mexico [15] The AUC for the SARC-F scores was calculated and ROC curve analysis was conducted. EWGSOP: European Working on Sarcopenia in Older People; PPV: positive predictive value; NPV: negative predictive value; AUC: area under the curve; SARC-F: Strength, Assistance walking, Rise from a chair, Climb stairs, and Falls

The mean SARC-F score in our population was 2.1±1.5 points, with a range of 0-7 points. This score was significantly higher in women versus men (3±2 vs 2±1, p <0.05). By applying the diagnostic cut-off point of SARC-F >4 points, 17% (n=16) of our population was classified as being at risk of sarcopenia (Figure 2A). The risk showed a statistical tendency to be more frequent in women (25.7% vs 11.9%; p = 0.09) (Table 2). 

When evaluating the performance of the SARC-F questionnaire for the diagnosis of sarcopenia as a continuous variable (questionnaire score), the ROC curve showed that its performance was poor in the overall population with an AUC of 0.40 (95%CI, 0.12-0.35, p = 0.35). The performance was equally poor in both male sex with an AUC of 0.44 (95%CI, 0.09-0.78, p = 0.66) and female sex with an AUC of 0.3 (95%CI, 0.01-0.60, p = 0.15) (Figures 2B, 2C).

When evaluating SARC-F as a dichotomous variable (>4 points vs. <4 points), its overall diagnostic performance was moderate to poor, with a sensitivity of 13% (95% CI, 2.2-47.1%), a specificity of 83% (95%CI, 73.2-89.1%), a positive predictive value (PPV) of 6% (95%CI, 1.1-28.3%), and a negative predictive value (NPV) of 91% (95%CI, 82.6-95.6%) (Figure 2C). Figure 2C illustrates the diagnostic performance of the SARC-F questionnaire stratified by sex.

When comparing the EWGSOP2 sarcopenia diagnostic criteria between patients with > or < 4 points on the SARC-F questionnaire, we observed that HGS (13 ± 5.8 vs 23.9 ± 9.3, p <0.01) and gait speed (0.6 ± 0.1 vs 0.9 ± 0.2, p < 0.01) were significantly lower in those patients with scores > 4 points. However, no significant differences were found in ASMMi (7.2 ± 1.1 vs 7.7 ± 1.2, p=0.15) (Table 3).

**Table 3:** Comparison of EWGSOP2 criteria between patients with SARC-F >4 vs <4 points. Diagnostic criteria for sarcopenia according to the EWGSOP2 [1]; Spanish version of the SARC-F adapted and validated for Mexico [15] The comparison between groups was performed using the X^2^ test and Student's t-test according to the data. SARC-F: Strength, Assistance walking, Rise from a chair, Climb stairs, and Falls; EWGSOP2: European Working Group on Sarcopenia in Older People

Variables	SARC-F < 4		SARC-F > 4		p / X^2^ or Student´s t-test values
	X	SD/%	X	SD/%	
Handgrip Strength (Kg)	23.9	9.3	13	5.8	<0.01 (t = 4.48)
Appendicular Muscle Mass Index (Kg/m^2^)	7.7	1.2	7.2	1.1	0.15 (t = 1.44)
Gait Speed (m/s)	0.97	0.21	0.6	0.18	<0.01 (t = 6.40)
Sarcopenia by EWGSOP2 Criteria					
Without Sarcopenia	71	91.0%	15	93.8%	1.0 (X^2^ = 0.12)
With Sarcopenia	7	9.0%	1	6.3%	
Handgrip Strength Criterion					
Normal handgrip strength	41	52.6%	2	12.5%	0.005 (X^2^= 8.58)
Low handgrip strength	37	47.4%	14	87.5%	
Appendicular Muscle Mass Criterion					
Normal Appendicular Muscle Mass	67	85.9%	15	93.8%	0.68 (X^2^ = 0.73)
Low Appendicular Muscle Mass	11	14.1%	1	6.3%	
Gait Speed Criterion					
Speed > 0.8 m/s	62	79.5%	2	12.5%	0.001 (X^2^ = 27.4)
Speed < 0.8 m/s	16	20.5%	14	87.5%	

According to the EWGSOP2 diagnostic cut-off points for sarcopenia, the criteria for reduced HGS (87.5% vs 47.4%) and slow gait speed (87.5% vs 20.5%) were significantly more frequent in patients with SARC-F > 4 points. In contrast, the criterion for low ASMMi was less frequent in these patients (6.3% vs 14.1%; p = 0.68) compared to patients with SARC-F < 4 points (Table 3).

When correlating the SARC-F questionnaire score with the ASMMi, HGS, and gait speed (Table 4), we observed that the SARC-F score had a moderate negative correlation with HGS (r= -0.386, p <0.05) and gait speed (r= -0.531, p <0.05). However, the correlation with ASMMi was poor and non-significant (r= -0.098, p = 0.34).

**Table 4:** Correlation matrix between SARC-F score and HGS, ASMMi, and gait speed. Pearson correlation coefficient, *p <0.05; Spanish version of the SARC-F adapted and validated for Mexico [15] ASMMi: appendicular skeletal muscle mass index; HGS: handgrip strength; SARC-F: Strength, Assistance walking, Rise from a chair, Climb stairs, and Falls

	SARC-F score	HGS	Gait Speed	ASMMi
SARC-F score	1	-.386^*^	-.531^*^	-.098
HGS	-.386^*^	1	.353^*^	.250^*^
Gait speed	-.531^*^	.353^*^	1	.128
ASMMi	-.098	.250^*^	.128	1

## Discussion

The objective of this study was to evaluate the utility of the SARC-F questionnaire as a screening tool for the diagnosis of sarcopenia in patients with ESRD on HD. Our results show that the SARC-F, using a cut-off point > 4, had moderate to poor performance, characterized by high specificity (83%) but low sensitivity (13%) and a poor AUC when evaluated as a continuous variable. This performance is consistent with findings from other studies in the HD population.

In a cross-sectional study conducted in Japan with 179 HD patients, Imamura et al. evaluated the validity of the SARC-F questionnaire as a screening tool for sarcopenia defined according to the AWGS 2019 criteria [13]. The prevalence of sarcopenia was 27.4%, and 33.0% of their patients had SARC-F >4. The instrument, similar to our study, showed low sensitivity (42.9%) and moderate specificity (70.8%) for detecting sarcopenia, with an AUC = 0.57. Similarly, Du et al. reported a SARC-F sensitivity of 48.7% and specificity of 89.5%, with an AUC of 0.73 for the detection of sarcopenia in HD patients [16]. Both studies conclude that in HD patients, it is recommended to combine the SARC-F with objective measures of muscle mass and performance [16].

The limited diagnostic capacity of the SARC-F, particularly its low sensitivity, has also been documented in other clinical contexts. The meta-analysis by Voelker et al., which consolidated data from over 15,000 participants, reported a high average specificity (90%) but low sensitivity (31%) and a combined AUC of 0.64 [17]. The authors conclude that the SARC-F is useful as an initial screening tool, especially for ruling out sarcopenia, but it does not replace instrumental assessments of muscle mass and strength.

A possible explanation for the limited performance of the SARC-F in patients on HD is that this questionnaire primarily evaluates functional limitations, such as strength, mobility, or falls, under the theoretical assumption that these limitations are exclusively due to a reduction in skeletal muscle mass. However, patients on HD present multiple additional conditions, such as obesity, heart failure, malnutrition, chronic inflammation, autonomic neuropathy, anemia, fluid overload, and electrolyte disturbances, that may contribute more significantly to functional limitations, independently of muscle mass. This could weaken the direct relationship between the questionnaire score and the formal diagnosis of sarcopenia according to the EWGSOP2 criteria. Our results may support this hypothesis: patients with SARC-F > 4 showed lower muscle strength and gait speed, but without significant differences in skeletal muscle mass compared to those with SARC-F < 4 score. Likewise, the SARC-F score presented a moderate negative correlation with handgrip strength and gait speed, but a poor correlation with the ASMMi (Table 4).

Our study has several limitations that should be acknowledged. First, it was conducted at a single center with a relatively small sample size, which may limit the external validity of our findings. Nonetheless, the results align with patterns previously reported in other HD cohorts. The low prevalence of sarcopenia observed in our population (8.5%), although lower than values described in the literature, may be partially explained by the strict exclusion criteria (e.g., patients with amputations or inability to walk), which may have resulted in the selection of a functionally healthier sample. Another limitation is the use of bioimpedance analysis for estimating muscle mass rather than dual-energy X-ray absorptiometry (DEXA), the gold standard, which could have led to an underestimation of sarcopenia prevalence. However, EWGSOP2 guidelines support the use of bioimpedance when DEXA is not available. Finally, we used the Sergi equation to estimate appendicular muscle mass, which was originally validated in a geriatric population and has not been validated in HD patients. Although all of these aspects could be considered limitations of our study, they actually reflect gaps in the current knowledge about sarcopenia in patients on HD, such as: What is the gold standard for assessing body composition in this population? What are the appropriate cut-off values for muscle strength and gait speed? Are these cut-off points universally applicable to all patients, across all races and all regions?

## Conclusions

The SARC-F questionnaire demonstrated limited sensitivity for early identification but acceptable specificity for ruling out sarcopenia in ESRD patients on HD. These performance highlight the need to adopt a composite screening approach that integrates objective measurements of muscle mass and physical performance in patients on, as well as the urgent validation of alternative screening tools with greater sensitivity for this specific population.
